# miR-181d and c-myc-mediated inhibition of CRY2 and FBXL3 reprograms metabolism in colorectal cancer

**DOI:** 10.1038/cddis.2017.300

**Published:** 2017-07-27

**Authors:** Xiaofeng Guo, Yuekun Zhu, Xinya Hong, Mukun Zhang, Xingfeng Qiu, Zhenfa Wang, Zhongquan Qi, Xuehui Hong

**Affiliations:** 1 Department of Gastrointestinal Surgery, Zhongshan Hospital of Xiamen University, Xiamen, Fujian, China; 2 Department of General Surgery, The First Affiliated Hospital of Harbin Medical University, Harbin, Heilongjiang, China; 3 Department of Medical Imaging and Ultrasound, Zhongshan Hospital of Xiamen University, Xiamen, Fujian, China; 4 Department of Oncology, Anhui Provincial Hospital, Hefei, Anhui, China; 5 Organ transplantation institute of Xiamen University, Fujian Key Laboratory of Organ and Tissue Regeneration, Xiamen, Fujian, China; 6 Institute of Gastrointestinal Oncology, Medical College of Xiamen University, Xiamen, Fujian, China; 7 Xiamen Municipal Key Laboratory of Gastrointestinal Oncology, Xiamen, Fujian, China

## Abstract

Colorectal cancer (CRC) is the second major cause of tumor-related deaths. MicroRNAs (miRNAs) have pivotal roles in CRC progression. Here, we describe the effect of miR-181d on CRC cell metabolism and underlying molecular mechanism. Our data firmly demonstrated that knockdown of miR-181d suppressed CRC cell proliferation, migration, and invasion by impairing glycolysis. Mechanistically, miR-181d stabilized c-myc through directly targeting the 3′-UTRs of CRY2 and FBXL3, which subsequently increased the glucose consumption and the lactate production. Inhibition of c-myc via siRNA or small molecular inhibitor abolished the oncogenic effects of miR-181d on the growth and metastasis of CRC cells. Furthermore, c-myc/HDAC3 transcriptional suppressor complex was found to co-localize on the CRY2 and FBXL3 promoters, epigenetically inhibit their transcription, and finally induce their downregulation in CRC cells. In addition, miR-181d expression could be directly induced by an activation of c-myc signaling. Together, our data indicate an oncogenic role of miR-181d in CRC by promoting glycolysis, and miR-181d/CRY2/FBXL3/c-myc feedback loop might be a therapeutic target for patients with CRC.

Colorectal cancer (CRC) was shown to be one of the most common causes of tumor-related deaths in the world with characteristics of fast progression, unfavorable curative effect, and poor prognosis.^1^ Previous studies showed over 600 000 deaths and 1.2 million new cases diagnosed each year.^2^ In spite of important advancements in diagnostic and therapeutic methods to CRC over the past ten years, cure rate of CRC remains insufficient due to its increasing morbidity and mortality, and the prognosis of patients with liver metastasis is still poor.^3^ Therefore, it was urged to make further exploration and search better way to get its morbidity and mortality under control.

It is well known that altered energy metabolism generally associated with uncontrolled proliferation of cancer cells.^4^ And it is becoming increasingly clear that tumor cells have special metabolic requirements to develop into three-dimensional tumor masses.^5^ There are two major pathways to maintain glucose homeostasis in mammalian cells: the anabolic gluconeogenesis pathway and the catabolic glycolysis/oxidative phosphorylation pathway.^6^ In the presence of oxygen, glucose in normal cells is first converted to pyruvate in the cytoplasm, and then pyruvate is used to produce the ATP via tricarboxylic acid cycle in the mitochondria.^7^ However, in tumor cells, pyruvate is preferentially converted to lactate even in the presence of oxygen, a process known as aerobic glycolysis or the Warburg effect.^7^ Increased rate of aerobic glycolysis and lactate production are not only typical metabolic characteristics of cancer cells, but also predictive factors of metastasis and overall survival of patients.^8^ However, molecular mechanisms underlying the Warburg effect in CRC remain unclear.

Recent studies demonstrate a critical role of microRNAs (miRNAs) in CRC growth and metastasis.^9^ miRNAs have also been shown to regulate metabolic pathways including the Warburg effect, by affecting the levels of key metabolic enzymes such as PKM2 and STAT3.^10^ MiRNAs are short non-coding RNAs between 21 and 25 nucleotides in length.^11^ By binding to the 3′-untranslated region (UTR) of various target mRNAs, miRNAs promote the degradation or translational repression of their targets, thus affecting multiple disease-related signal pathways.^11^ Therefore, identifying therapeutic miRNAs would be of great clinical value. Recently, miR-181 family has been found to critically affect tumor growth via glycolysis metabolism. However, effects of miR-181 family members (including a, b, c, d) on tumors are totally divergent. For example, miR-181a/b was reported to be dysregulated, and acted as either an oncogene or a tumor-suppressor gene via affecting metabolic shift.^12^ However, little was known about the clinicopathological relevance of miR-181d in CRC. Especially, so far, there is no report about the role of miR-181d in tumor metabolism. Thus, in this study, we aimed to explore the association between the expression of miR-181d and glycolysis in CRC, and to evaluate its value in prognosis of this tumor.

## Results

### The level of miR-181d is upregulated in CRC and significantly correlates with tumor growth and metastasis

**Figure 1:**
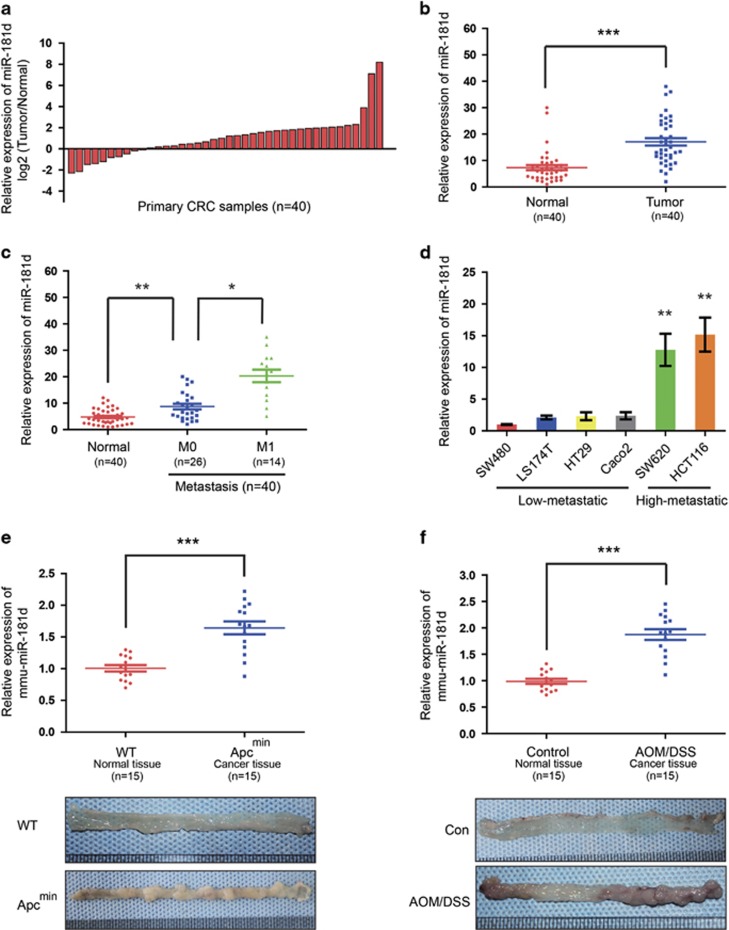
**T**he level of miR-181d is frequently enhanced in CRC tissues and cell lines. (**a** and **b**) qRT-PCR was used to measure the level of miR-181d expression in human CRC tissues and corresponding normal tissues from 40 patients with CRC. (**c**) The relative of miR-181d expression with CRC metastasis. (**d**) qRT-PCR was used to analyze the level of miR-181d in CRC cell lines with different metastatic potentials. (**e** and **f**) qRT-PCR analysis of miR-181d expression (top) and representative pictures of colon tissues (bottom) in Apc^Min^ mice (*n*=15) (**e**), or in AOM/DSS-treated mice (*n*=15) (**f**), and their controls. For the above qRT-PCR data, relative quantification was achieved by normalization to the amount of snRNA U6

miR-181a/b has been reported to function as a tumor suppressor and an oncogene,^12^ but very little is known about the role of miR-181d in tumor. To investigate the clinicopathological significance of miR-181d in CRC, we first studied miR-181d expression patterns in 40-paired human CRC tissues and corresponding normal colorectal tissues. As indicated in Figure 1a, compared with the normal counterparts, the level of miR-181d was significantly upregulated in 30 of the 40 (75%) CRC tissues. Compared with their controls, miR-181d expression was increased by nearly threefold in the CRC tissues (Figure 1b). Clinicopathologic relevance analysis indicated that miR-181d expression was positively correlated with CRC metastasis (Figure 1c). In consistency with the aforementioned data, it was observed that miR-181d expression was much higher in highly metastatic human CRC cell lines (HCT116 and SW620) than that in CRC cell lines with low-metastatic potential (SW480, LS174T, HT29, and Caco-2) (Figure 1d). The findings suggest that that miR-181d might act as an oncomiR and increased miR-181d expression might contribute to CRC malignancy.

Next, to further elucidate the function of miR-181d in CRC development, two mouse CRC models, azoxymethane/dextran sodium sulfate (AOM/DSS) mice and APCMin mice were analyzed. It was found that the level of miR-181d in CRC tumors from APCMin mice was significantly higher than that in control tissues from wild-type mice (Figure 1e). Similarly, compared with that in the control tissues, mice treated with AOM/DSS showed a significantly enhanced level of miR-181d in CRC samples (Figure 1f). Collectively, our data indicate that the level of miR-181d is not only upregulated in clinical CRC specimens, cell lines, and mouse models, but also positively correlated with the progression of CRC.

### Knockdown of miR-181d inhibits the proliferation, colony formation, and metastasis of CRC cells *in vitro* and *in vivo*

**Figure 2:**
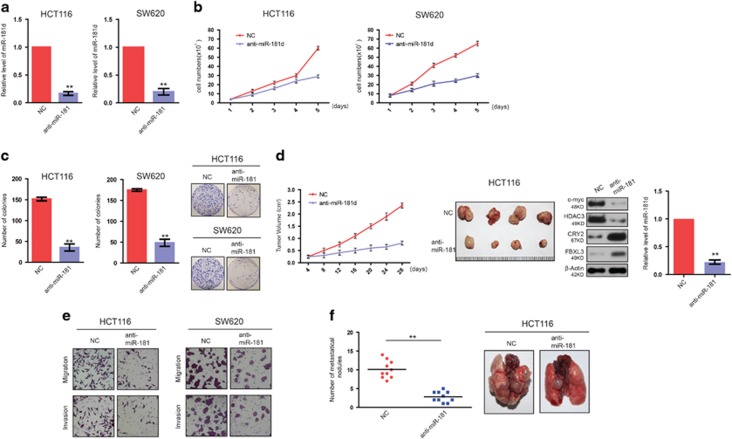
Knockdown of miR-181d inhibits the proliferation, colony formation, and metastasis of CRC cells *in vitro* and *in vivo*. (**a**) Effective knockdown of miR-181d in CRC cells was confirmed by qRT-PCR. (**b**) After miR-181d knockdown, proliferation of CRC cells was measured by CCK8 assay. (**c**) Colony formation ability of CRC cells was suppressed once silencing miR-181d. (**d**) Analysis of the xenograft tumors formed by CRC cells with or without miR-181d knockdown (first and second panel). CRY2, FBXL3, c-myc, HDAC3 (third panel), and miR-181d levels (fourth panel) were presented. 6 S rRNA and actin were used as internal controls of qRT-PCR and western blotting, respectively. (**e**) Migration and invasion assays of CRC cells after miR-181d knockdown. (**f**) Assay of lung metastatic foci number after tail vein injection and representative images

To investigate the function of miR-181d, we stably knocked down the expression of miR-181d in HCT116 and SW620 cells. Knockdown efficiency was confirmed by qRT-PCR (Figure 2a). It was observed that after miR-181d knockdown, proliferation of CRC cells was significantly inhibited (Figure 2b). Colony formation assays confirmed that silencing endogenous miR-181d greatly suppressed the colony formation ability in CRC cells (Figure 2c), which was further verified using xenograft mice models. We noted that compared with the control groups, the inhibition of miR-181d in HCT116 cells significantly decreased subcutaneous tumor growth (Figure 2d). These data indicate that miR-181d promotes the tumor growth of CRC cells *in vivo*.

Next, we investigated the effect of miR-181d on the migratory and invasive abilities of CRC cells. As shown in Figure 2e, the suppression of endogenous miR-181d dramatically decreased the migration and invasion of CRC cells. We then established the lung metastasis model through tail vein injection to further confirm the role of miR-181d in CRC metastasis *in vivo*. Results showed that mice bearing HCT116 cells with miR-181d-knockdown had less lung metastatic nodules than control mice (Figure 2f). Collectively, these observations demonstrate that miR-181d promotes the tumor growth and metastasis of CRC cells.

### The inhibition of miR-181d decreases glycolysis in CRC cells

**Figure 3:**
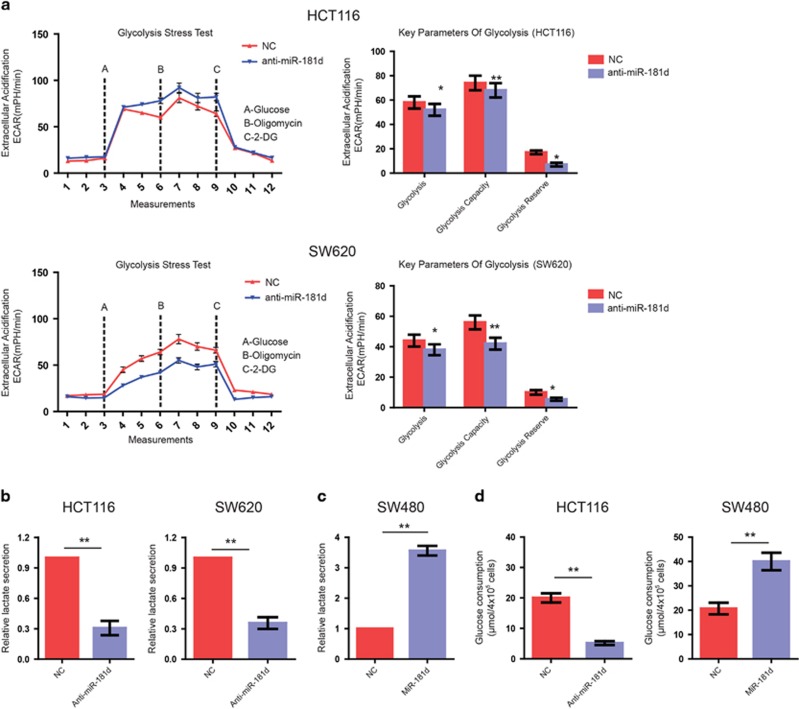
miR-181d promotes CRC cell glycolysis. (**a**) Following indicated treatment, ECAR was measured by the Glycolysis Stress test in CRC cell lines. And the glycolysis under basal conditions, the glycolytic capacity, and the glycolytic reserve were analyzed. (**b** and **c**) Lactate production in CRC cells transfected with anti-miR-181d or miR-181d mimics was measured by lactate assay. (**d**) Forced expression of miR-181d increased the consumption of glucose while the knockdown of miR-181d had an opposite effect

Given that aerobic glycolysis is a pivotal mechanism for CRC cells to sustain aggressive features such as proliferation and invasion,^13^ we next explore the role of miR-181d in CRC cell glucose metabolism. First, we performed glycolysis stress test. As shown in Figure 3a, after treatment with glucose or oligomycin, anti-miR-181d-transfected CRC cells exhibited lower levels of the extracellular acidification rate (ECAR) compared with the control. Moreover, miR-181d knockdown was also found to decrease the glycolysis under basal conditions, the glycolytic capacity and the glycolytic reserve in CRC cells (Figure 3a).

Another well-characterized metabolic alteration of glycolysis is a high lactate production, opposed to carbon dioxide, as the final metabolite.^14^ To further confirm the effect of miR-181d on the Warburg effect, levels of lactate production were measured in CRC cells. After miR-181d was inhibited, the level of lactate in the culture media was obviously decreased in CRC cells (Figure 3b). On the contrary, the level of lactate was significantly increased in CRC cells once miR-181d overexpression (Figure 3c). In parallel, forced expression of miR-181d elevated the consumption of glucose while silencing miR-181d established an opposite effect (Figure 3d). Taken together, these results suggested that miR-181d promoted the glycolysis of CRC cells.

### C-myc is required for the metabolic shift induced by miR-181d

**Figure 4:**
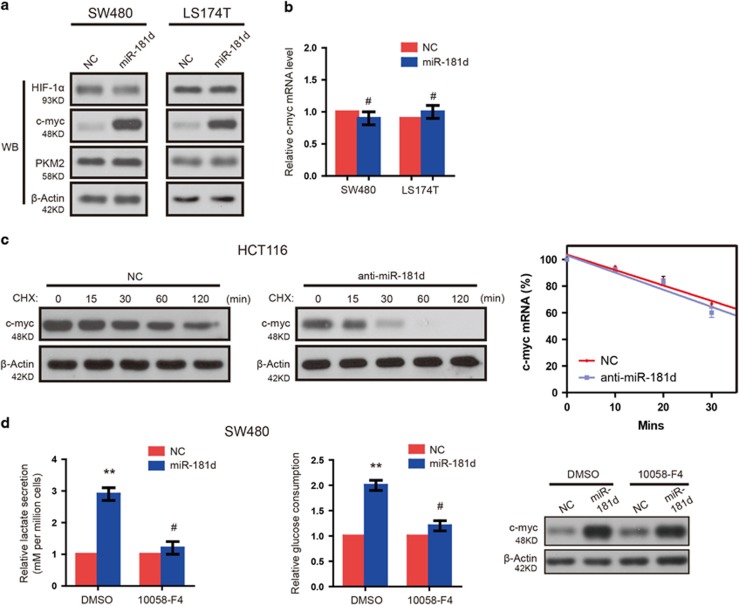
c-myc is required for the miR-181d-induced metabolic shift. (**a**) Western blot assays show that miR-181d increases c-myc protein, not mRNA levels, and does not affect total PKM2, or HIF1*α* expression. (**b**) The effect of miR-181d overexpression on c-myc mRNA levels in CRC cells. Relative quantification was achieved by normalization to the amount of GAPDH (for mRNAs). (**c**) After cycloheximide treatment, western blot was used to analyze the level of c-myc protein in CRC cells with indicated plasmids at the indicated times(left and middle panel). And c-myc mRNA stability were determined using actinomycin D (ActD) mRNA stability assays in HEK293T cells(right panel). c-myc mRNAs was quantified relative to 18 S rRNA mRNA. (**d**) Inhibition of c-myc with 10058-F4 blocked miR-181d-induced increases in lactate secretion and the rate of glucose consumption(left and middle panel). And c-myc expression was analyzed via western blot with actin as loading control(right panel)

Previous reports have demonstrated that c-myc, hpoxia inducible factors *α* (HIF1*α*), and PKM2 play central roles in regulating cell glycolysis.^15^ Thus, we examined the effect of miR-181d on their levels in CRC cells. We found that miR-181d expression increased significantly c-myc protein levels whereas it did not change its mRNA level in SW480 and LS174T cells (Figure 4a). However, western blot and qRT-PCR analysis revealed no differences in the protein and mRNA (data not shown) expression of HIF1*α* and PKM2 between miR-181d-overexpressing CRC cells and controls (Figure 4a). And miR-181d overexpression in CRC cells also has little effect on the mRNA levels of c-myc (Figure 4b). These findings strongly suggest that miR-181d modulates post-transcriptional regulation of c-myc. To determine whether the c-myc protein half-life was increased in anti-miR-181d-transfected cells, we examined c-myc protein turnover after addition of the translation inhibitor cycloheximide. As expected, c-myc had a very short half-life in anti-miR-181d-transfected cells, which was evidenced by the rapid turnover within 15–30 min, while c-myc was stabilized in the presence of cycloheximide (Figure 4c). The above findings were further confirmed using c-myc mRNA stability assays (Figure 4c). Notably, re-introducing miR-181d in these cells increased the half-life of endogenous c-myc (data not shown). Thus, we hypothesized that miR-181d would stimulate CRC cell glycolysis by increasing c-myc stability. Consistent with this prediction, 10058-F4, a c-myc inhibitor,^16^ significantly inhibited the increased rate of glucose and lactate production observed in miR-181d-overexpressing cells (Figures 4c and d). These data suggest that c-myc is required for miR-181d-induced changes in glycolysis.

### Inhibition of the Warburg effect by anti-miR-181d, or c-myc inhibitor, or LDH inhibitor leads to decreased cell proliferation and invasion in CRC cells

**Figure 5:**
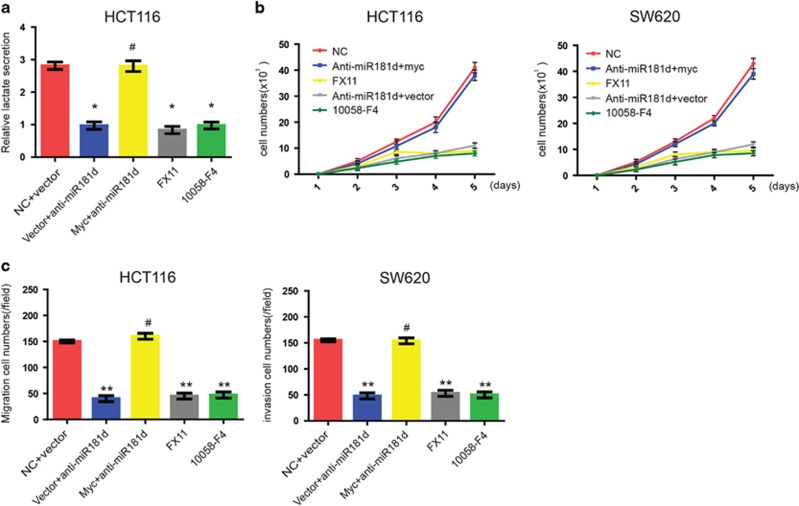
Inhibition of the Warburg effect by FX11, or anti-miR-181d, or 10058-F4 significantly reduced CRC cell proliferation and metastasis. (**a**) The effects of indicated treatment on the lactate production in CRC cells. (**b**) Effect of indicated treatment on the proliferation of CRC cells. (**c**) Effect of indicated treatment on the metastasis of CRC cells

To confirm that miR-181d promotes CRC growth and metastasis by enhancing c-myc-mediated glycolysis, we compared the effects of anti-miR-181d, 10058-F4, and FX11 (a lactate dehydrogenase A (LDHA, which executives the final step of Warburg effect by converting the pyruvate to lactate) inhibitor) on CRC cells.^17^ LDHA executives the final step of Warburg effect by converting the pyruvate to lactate.^17^ As shown in Figure 5a, anti-miR-181d, or 10058-F4, or FX11 significantly inhibited lactate content, while c-myc transfection rescued the phenotype of anti-miR-181d in CRC cells. Subsequently, proliferation and migration of CRC cells were analyzed in different-treated cells to evaluate the effects of glycolysis inhibition. CCK8 assays indicated that proliferation of CRC cells was inhibited to a similar degree by FX11, or anti-miR-181d, or 10058-F4 while c-myc reintroduction reverted the effect of anti-miR-181d (Figure 5b). Notably, FX11, or anti-miR-181d, or 10058-F4 also significantly decreased CRC cell migration and invasion abilities to a similar degree in CRC cells (Figure 5c). These data indicated that upregulation of miR-181d in CRC promoted glycolysis, which may be mediated by c-myc and responsible for aggressiveness of CRC cells.

### FBXL3 and CRY2 are dNirect targets of miR-181d in CRC cells

**Figure 6:**
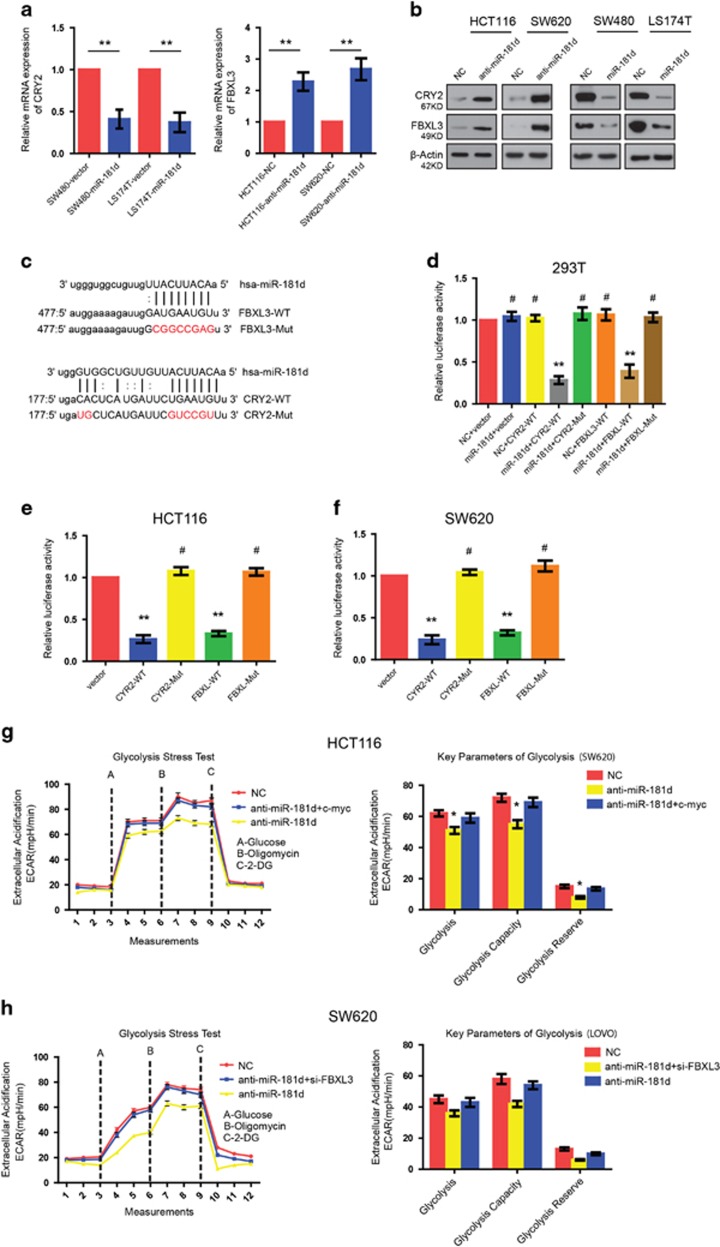
FBXL3 and CRY2 are direct targets of miR-181d. (**a**) qRT- PCR analysis of FBXL3 and CRY2 mRNA expression. (**b**) The expression of FBXL3 and CRY2 was analyzed using western blot in the indicated cells. (**c**) Predicted miR-181d target sequences in the 3′-UTRs of FBXL3 and CRY2, and their mutations were generated in the 3′-UTR sequences at the complementary sites for the seed regions in miR-181d. (**d**) Luciferase reporter assays of the indicated cells. (**e,f**) Luciferase assays with the endogenous levels of miR-181d in HCT116 and SW620 cell lines. (**g**,**h**) The effects of FBXL3 Knockdown and c-myc overexpression on miR-181d-induced glycolysis in CRC cells

Although the above data suggest that miR-181d decides the stability of c-myc and c-myc mediates the effect of miR-181d on glycolysis in CRC cells, c-myc is not a direct target of miR-181d. To further reveal the mechanism of miR-181d-induced glycolysis, it is necessary to determine its direct mRNA targets that might mediate the role of miR-181d in the growth and metastasis of CRC. Based on binding sites in the 3′-UTR, three different mRNA target-predicting algorithms (TargetScan, Pictar, and miRANDA) were used to predict potential direct targets of miR-181d. The results indicated that FBXL3 and CRY2 were theoretical target genes of miR-181d (Figure 6c). Given that FBXL3 and CRY2 had been recently reported to ubiquitinate and degrade c-myc cooperatively,^18^ FBXL3 and CRY2 were chosen for further investigation. Western blot and real-time PCR analyses indicated that the mRNA and protein levels of FBXL3 and CRY2 were significantly downregulated in cells with miR-181d overexpression, whereas they were upregulated after inhibition of miR-181d (Figures 6a and b).

To confirm that FBXL3 and CRY2 were directly inhibited by miR-181d, we subcloned the FBXL3 and CRY2 3′-UTR fragments containing miR-181d-binding site and their mutant fragments into the luciferase report construct. Dual-luciferase reporter assays showed that co-expression of miR-181d markedly inhibited the luciferase reporter activity of the wild-type FBXL3 and CRY2 3′-UTR, while the inhibitory effects were abolished when the putative miR-181d seed-binding regions were mutated (Figure 6d). In addition, luciferase reporter analyses with the endogenous levels of miR-181d in HCT116, and SW620 cells also demonstrated that miR-181d inhibited FBXL3 and CRY2 expression via binding to their 3′-UTR (Figures 6e and f). Furthermore, knockdown of either FBXL3 or CRY2 abolished the increase of c-myc induced by miR-181d in CRC cells (data not shown). In addition, knockdown of FBXL3 or c-myc overexpression abolished anti-miR-181d-mediated glycolysis inhibition (Figures 6g and h). Therefore, our data demonstrate that FBXL3 and CRY2 are direct targets of miR-181d.

### C-myc promotes miR-181d upregulation while inhibits the expression of CRY2 and FBXL3 in CRC cells

**Figure 7:**
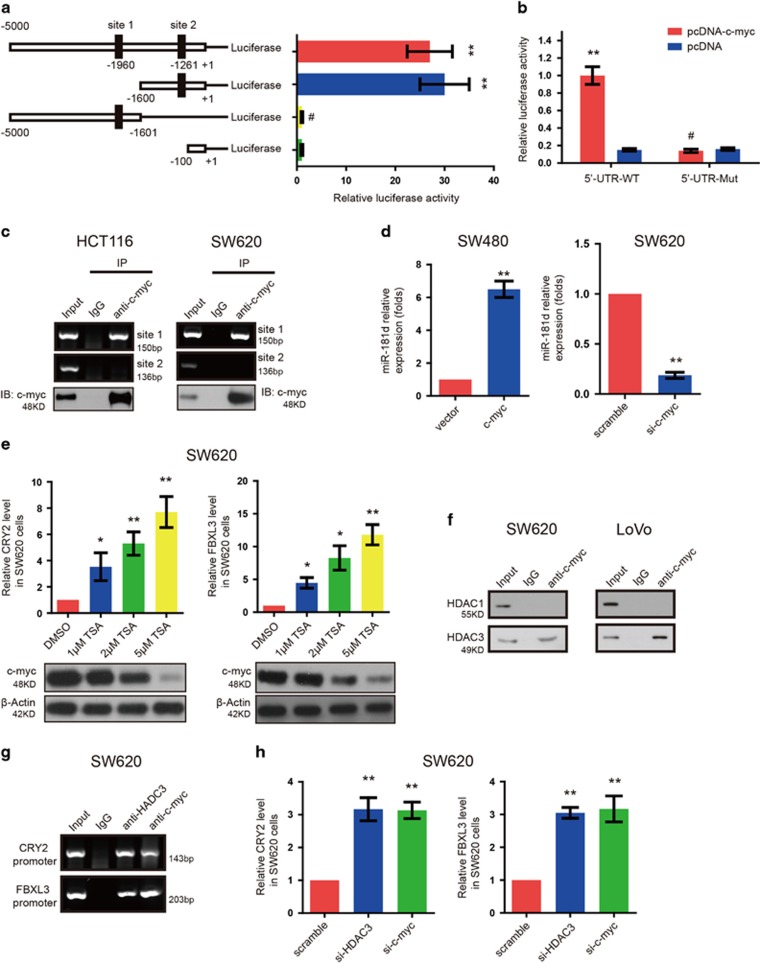
c-myc promotes miR-181d upregulation while inhibits the expression of CRY2 and FBXL3 in CRC cells. (**a**) Schema of miR-181d promoter-containing pGL3-luciferase reporter constructs. Sites 1 and 2 are two potential c-myc-binding sites. (**b**) Enforced expression of c-myc inhibits the activity of wild-type, not mutant miR-181d promoter. The dual-luciferase reporter assay was performed in HEK-293 cells. (**c**) ChIP data indicate that c-myc binds on the c-Myc-binding site at -1261 bp upstream of the miR-181d promoter in CRC cells and corresponding c-myc western blot-IP was shown. (**d**) si-c-myc, or c-myc expression plasmid, or empty vector was transfected into CRC cells. And miR-181d expression was analyzed by qRT-PCR. (**e**) TSA treatment significantly induces expression of CRY2 and FBXL3 in the CRC cells in a dose-dependent manner(upper panel). And c-myc levels were analyzed with western blot (lower panel).(**f**) Co-IP assay indicates that c-myc can interact with HDAC3, but not HDAC1, in CRC cells. (**g**) ChIP shows that HDAC3 binds on the same location of CRY2 and FBXL3 promoter with c-myc. (**h**) Inhibition of HDAC3 by si-HDAC3 transfection dramatically accelerates expression of CRY2 and FBXL3 in CRC cells

To further elucidate the transcriptional regulation of miR-181d, we used miRstart and TransFac software to analyze the –5000 bp sequences in miR-181d gene promoter. Upstream of the pre-miR-181d sequences, two putative E box elements for c-myc binding were identified. Then, a series of reporter plasmids containing different fragments within the miR-181d promoter were made to determine the effect of c-myc on miR-181d expression. Consequently, the reporter construct without c-myc-binding site had no response to c-myc, while only the reporter gene containing one c-myc-binding site, which is located at −1261 bp upstream of miR-181d, significantly activated miR-181d transcription when c-myc was co-transfected into the cells (Figure 7a). These results were strengthened by luciferase reporter assay which enforced that c-myc could markedly increase the miR-181d promoter activity in 293T cells, while mutant of the c-Myc-binding site abolished this effect (Figure 7b). Chromatin immunoprecipitation (ChIP) assay further confirmed that this c-myc-binding site in the miR-181d promoter effectively binds to c-myc protein in CRC cells (Figure 7c). Moreover, the western blot showed that the expression of miR-181d in CRC cells with c-myc overexpression was indeed higher than that in control cells, while knockdown of c-myc in both CRC cells by siRNAs transfection significantly decreased expression of miR-181d (Figure 7d). All these results fully indicated that c-myc could positively regulate miR-181d expression.

As reported, c-Myc can recruit HDAC1 or HDAC3 to inhibit expression of specific genes,^19^ which lead us to investigate whether the c-myc/HDAC1/3 complex was involved in the transcriptional repression of CRY2 and FBXL3. To address this hypothesis, we initially examined effects of deacetylase inhibitor Trichostatin A (TSA) on CRY2 and FBXL3 expression. As shown in Figure 7e, TSA caused a dose-dependent increase of CRY2 and FBXL3 expression in CRC cells while c-myc levels oppositely changed, suggesting the role of HDACs in CRY2 and FBXL3 expression. Our co-immunoprecipitation (Co-IP) results indicated that HDAC3, but not HDAC1, physiologically interacted with c-myc in CRC cells (Figure 7f). ChIP-PCR assay showed that HDAC3 could co-localize with c-myc to the same promoter region of CRY2 and FBXL3 (Figure 7g). And inhibition of HDAC3 by siRNAs could increase CRY2 and FBXL3 expression in CRC cells (Figure 7h). All these data indicated that c-myc blocks the transcription of FBXL3 and CRY2 in CRC cells, suggest a feedback loop established. This feedback loop was further confirmed by c-myc overexpression or knockdown of CRY2 and FBXL3 experiments in SW480 cells (Supplementary Figure 1).

## Discussion

In this study, we demonstrated that miR-181d was significantly upregulated in human CRC tissues and cell lines compared with their controls. We also found that high expression of miR-181d can promote cell proliferation, migration, and invasion, strongly suggesting that miR-181d is a prognostic indicator of CRC cell growth and metastasis and acts as an oncomiR in CRC. Our clinicopathological assays showed that miR-181d dysregulation associated with CRC metastasis and TNM stage. To explore miR-181d targets that might explain its oncogenic role in CRC, we identified CRY2 and FBXL3 as two direct targets of miR-181d based on the following evidence that (i) miR-181d overexpression significantly decreased CRY2 and FBXL3 protein expression; (ii) CRY2 and FBXL3 and miR-181d expression levels were negatively correlated; and (iii) 3′-UTR-luciferase reporter activities of both CRY2 and FBXL3 were suppressed by miR-181d overexpression, although this effect was not observed after mutating the miR-181d seed-binding sequence. In addition, we also demonstrated that miR-181d promoted aerobic glycolysis by protecting c-myc from FBXL3 and CRY2-mediated degradation, which is responsible for CRC growth and metastasis. Therefore, we suggest that there is an association between miR-181d and the Warburg effect in CRC.

The reprogramming in energy metabolism has recently been listed as 1 of the 10 hallmarks of cancer.^20^ Even though in the presence of ample oxygen, cancer cells also display high levels of glycolysis, which is critical for tumor cell proliferation and invasion.^21^ Therefore, it has been pointed out that targeting glycolysis may provide a selective mechanism by which to specifically kill cancer cells, while avoiding injury to normal cells.^22^ Several potential candidates that are overexpressed and contribute to glycolysis in certain cancer types include LDHA, PKM2, HIF1*α*, STAT3, Glut1, and HK2.^23^ However, few studies on glucose metabolism in CRC cells have been reported. Here, we found that miR-181d was essential for CRC cell glycolysis. The inhibition of miR-181d by siRNA significantly decreased the level of the glycolysis under basal conditions, the glycolytic capacity, and the glycolytic reserve. To the best of our knowledge, this is the first report that miR-181d exerted its oncogenic roles by promoting the Warburg effect. Supporting our data, overexpression of miR-181a also enhanced cell proliferation through increased glycolysis via affecting the PTEN/AKT pathway in CRC cells.^24^ However, contrary to our data, miR-181c inhibited glycolysis by targeting hexokinase 2 in cancer-associated fibroblasts.^25^

Although mice harboring genetically disrupted clock gene exhibit altered rates of tumor formation,^26^ the relationship between CRC and clock gene is not well understood and remains controversial. It is known from the literature that CRY2/FBXL3 is able to decrease the expression of c-myc via driving proteolytic turnover of T58-phosphorylated c-myc.^18^ However, this signaling pathway did not completely reveal either crosstalk between c-myc and CRY2/FBXL3, or the correlation of CRY2/FBXL3 with glycolysis and miRNA. Here, for the first time, we showed that both miR-181d and c-myc could directly regulate the expression of CRY2/FBXL3. Importantly, all of these molecules are key in our hypothesis model. Given that clinically targeting circadian gene or c-myc was either unsuccessful or impossible, this discovery is of great importance since there are many studies, including pre-clinical ones, testing the effect of miRNA inhibitors alone, or in combination with radiotherapy, chemotherapy, and immunotherapy in tumor treatment.^27^

In summary, we identified a c-myc-miR-181d-CRY2/FBXL3 feed-forward loop linking overexpression of miR-181d and c-myc with CRY2/FBXL3 suppression in CRC glucose metabolism. Based on our data, miR-181d acts as an oncomiR that is upregulated in CRC tissues and may significantly affect clinical outcome. These findings represent a novel potential approach for silencing c-myc/miR-181d signal pathway in CRC treatment.

## Materials and Methods

### Human CRC tissue specimens and cell lines

Fresh CRC tissue and paired adjacent non-tumor colorectal tissues were derived from 40 CRC patients who underwent initial surgery in the Department of Colorectal Surgery of the First Affiliated Hospital of Harbin Medical University between Jan 2013 and Jan 2015. The matched clinical information was collected and analyzed. All patients provided written informed consent according to the Helsinki Declaration. And the institutional ethics and scientific committee approved this study. The disease stage was determined according to the TNM classification scheme. The specimens were obtained after surgical resection and immediately frozen at −80 °C until use.

All CRC cell lines (SW620, HT29, HCT116, SW480, Caco-2, and LS174T) and the human 293T embryonic kidney cell line were obtained from the Department of Cell Biology at China Medical University. 293T, SW620, LS174T, and HT29 cells were cultured in DMEM supplemented with 10% fetal bovine serum. HCT116 cells were maintained in McCoy’s 5 A media supplemented with 10% fetal bovine serum. Caco-2 and LS174T cells were maintained in RPMI1640 media supplemented with 10% fetal bovine serum. All cells were cultured in a humidified atmosphere containing 5% CO2 at 37 °C.

### Migration and invasion assay

The migratory and invasive ability of cells were measured in 24-well transwell plates (Corning Costar, Tewksbury, MA, USA). For the cell migration assays, 1  ×  105 CRC cells with indicated treatment in 200 *μ*l serum-free medium were seeded into the top chamber of each insert, and 600 *μ*l of medium supplemented with 10% fetal bovine serum was added into the lower chambers. After 24 h of incubation at 37 °C, cells in the top chambers were carefully cleaned and fixed with 4% paraformaldehyde. Cells in the under chambers were removed by cotton swabs, stained with 0.1% crystal violet, and dried in the air. The number of migrating cells was evaluated in five independent fields under a microscope. For the invasion assay, chamber inserts were scrawled with 200 mg/ml of Matrigel (BD Biosciences, San Jose, CA, USA), dried 4 h at 37 °C, and 1  ×  105 cells were placed in the top chamber. Three independent experiments were performed.

### Western blot analysis

Clinical samples and cells were lysed in RIPA lysis buffer containing protease inhibitor. Cell lysates were separated by 10% sodium dodecyl sulfate–polyacrylamide gel electrophoresis and transferred to a polyvinylidene difluoride membrane (Millipore, Billerica, MA, USA). The membranes were blocked with 5% nonfat milk and incubated with indicated antibodies. A secondary antibody was then incubated with the membrane for 1.5 h. Signals were detected using the ECL detection reagent. The band densities of specific proteins were quantified after normalization with the density of GAPDH or Tubulin.

### Chromatin immunoprecipitation

ChIP was performed using EZ ChIP kit (Millipore) according to the instruction of the manufacturer. Briefly, CRC cells for each immunoprecipitation reaction were collected and cross-linked with 1% formaldehyde for 10 min at room temperature. Immunoprecipitation was performed after incubation with anti-c-Myc or anti-HDAC3 (Bio-Rad, Hercules, CA, USA) or IgG antibody (Bio-Rad) and subsequent incubation with Protein A agarose (Roche, Madison, WI, USA) overnight. After the DNA purification, PCR was used to amplify were used as templates to amplify the target sequences from the input and the immunoprecipitated DNA samples. The primers specific to miR-181d gene promoter were as follows: site 1: 5′-AGCTTGCATGTGGTCTGTGCACCT-3′ and 5′-CCAACAGCCACATGCACAAGA-3′ site 2: 5′-CCCAACCCCATGGAGGTATGATTACT-3′ and 5′-TTAGAGGGGCCAGCTGTAGTCTACA-3′. The primers specific to CRY2 and FBXL3 gene promoter were as follows: CRY2: 5′-TGAGACCGTCTACGTAAGGAGATG-3′ and 5′-TGACTTGCAAGACTGACAGCTGAC-3′ FBXL3: 5′-CCTTTACTTTCCCCATTGCGTTTC-3′ and 5′-GCCAAGGAAATGTACCAGAATAGTGAGAG-3′.

### Reverse transcription-PCR and real-time quantitative PCR

Total RNA was extracted with Trizol (Invitrogen, Grand Island, NY, USA). cDNA was amplified under the standard conditions. MiRNAs were reverse-transcribed using the TaqMan miRNA reverse transcription kit (Applied Biosystems, Grand Island, NY, USA) and miRNA-specific primers (Applied Biosystems). MiRNA expression levels were then analyzed using the TaqMan MicroRNA assay (Applied Biosystems) according to the manufacturer’s instructions. Quantitation of a ubiquitously expressed miRNA (U6) was performed as an endogenous control. The primers used for RT-PCR analysis were listed as followed: FBXL3: 5′-AGTGACAACGTCGAGCACAG-3′ and 5′-CGGTCGCTACCATTACCAGT-3′ CRY2: 5′-CCAGAGACGGGAAATGTTCTT-3′ and 5′-GCTTCATCCACATCGGTAACTC-3′ GAPDH: 5′-TGAAGGTCGGAGTCAACGGATTTGGT-3′ and 5′-CATGTGGGCCATGAGGTCCACCAC-3′miR-181d:5′-CCGCTCGAGAACTTGCCAAGGGTTTGGGGGAACA-3′ and 5′-CCGGAATTCATGTTCATCTACCAGTTTGCCCACT-3′ snRNA U6: 5′-TCGCTTCGGCAGCACATA-3′ and 5′-TTTGCGTGTCATCCTTGC-3′. For real-time quantitative PCR, cDNA was amplified under standard conditions. FBXL3 and CRY2 and miR-181d levels were respectively normalized to the expression of GAPDH and snRNAU6. The data were analyzed by deltaCt method.

### Oligonucleotides, cell transfection, and drug treatment

The miR-181d mimics, anti-miR-181d, si-FBXL3, si-CRY2, si-c-myc, si-HDAC3, and their negative controls were purchased from Dharmacon (Austin, TX, USA) and transfected into CRC cells with DharmFECT1 (Dharmacon) at a final concentration of 100 nM. After 6 h, the medium was changed and cells were collected at indicated time points for analyses. TSA (HDACs inhibitor) and 10058-F4 (c-myc inhibitor) were purchased from Sigma (St. Louis, MO, USA).

### Glycolysis stress test and glucose consumption

The ECAR was detected by the Seahorse XF96 Analyzer Glycolysis. The ECAR represents non-glycolytic acidification, which comprises CO2 evolution followed by its hydration to carbonic acid and bicarbonate, as well as proton extrusion. As glycolysis occurs, the resulting acidification from the medium around the cells is detected directly by the XF Analyzer and then reported as the ECAR. Briefly, cells are incubated in glycolysis stress test medium without glucose. A saturating concentration of glucose was firstly injected. Glucose is consumed by the cells and catabolized to lactate, leading ATP and protons production, with a corresponding rapid increase in ECAR. This glucose induced response is reported as the rate of glycolysis under basal conditions. The second injection is oligomycin. Oligomycin suppresses mitochondrial ATP production and thus shifts the energy production to glycolysis, with the increase in ECAR revealing the maximum glycolytic capacity. 2-DG, a glucose analog, which impairs glycolysis through competitive binding to glucose hexokinase, is the last injection. The subsequent decrease in ECAR further demonstrates that the ECAR produced in the experiment is from the glycolysis. The gap between the Glycolytic Capacity and Glycolysis Rate represents the Glycolytic Reserve. Glucose consumption was calculated by deducting the measured glucose concentration in the media from the original glucose concentration. Glucose levels were determined by the glucose assay kit (Sigma-Aldrich, St. Louis, MO, USA).

### Measurements of lactate production

The colorimetric lactate assay kit (Bio Vision, Milpitas, CA, USA) was used to measure lactate production according to the manufacturer’s instructions. CRC cells with miR-181d overexpression or knockdown were incubated in lactate assay buffer containing enzyme and lactate probes. Then, optical density was measured at 570 nm.

### mRNA stability assays

c-myc mRNA stability were determined using actinomycin D (ActD) mRNA stability assays in HEK293T cells. c-myc mRNAs was quantified relative to 18 S rRNA mRNA, at various times after addition of Actinomycin D (ActD; 6.5 *μ*g/ml; Sigma-Aldrich) to the cell culture medium. mRNA degradation was analyzed by quantitative RT-PCR as described above. The primers used for real-time PCR were:c-myc primers (forward: 5′-TGAGGAGACACCGCCCAC-3′ and reverse: 5′-CAACATCGATTTCTTCCTCATCTTC-3′); 18 S rRNA: (forward: 5′-GTAACCCGTTGAACCCCATT-3′ and reverse: 5′-CCATCCAATCGGTAGTAGCG-3′). The relative amount of c-myc mRNA at each time point was determined in three independent experiments by cross-normalization using the internal controls and setting the input amount of c-myc mRNA to 100%.

### Cell proliferation assay

CRC cells were seeded in 96-well culture plates (2 × 10^3^ cells/well) overnight. Cells were treated as indicated. Cell proliferation rate was determined using the CCK-8 kit (Beyotime Institute of Biotechnology, Jiangshu, China) according to the manufacturer’s instructions.

### miRNA reporter luciferase assay

Indicated cells were seeded into a 24-well plate and co-transfected with miR-181d, or control, or 3′-UTR-luciferase plasmids. The cells were collected at 48 h post-transfection, and the luciferase activity was measured by the Dual-Glo Luciferase Assay System (Promega, Madison, WI, USA).

### Colony formation assay

Indicated cells were plated in six-well plates at the density of 300 cells per well and maintained in medium for 12–20 days. The cells were washed with PBS, then fixed in methanol for 15 min and stained with crystal violet for 15 min. After that The plates were photographed.

### Animal models

BALB/c mice aged 4 weeks were purchased from SLAC laboratory animal company (SCXK-2007-004, Shanghai, China) and housed in a specific pathogen-free environment. Before the experiments began, the animals were allowed to acclimate to the housing facilities for 7 days. Animal experiments were approved by the Experimental Animal Ethical Committee of Xiamen University and performed in accordance with the P. R. China legislation on the use and care of laboratory animals. To demonstrate that miR-181d could promote CRC tumor growth and metastasis *in vivo,* we firstly transfected HCT116 cells with the lentiviral plasmids for expression of anti-miR-181d or NC (Negative control). These plasmids were LV-3 (pGLVH1/GFP+Puro) purchased from Genepharma. After infection, these cells were selected using puromycin. Then HCT116 cells (1 × 10^6^) with matrigel (BD Biosciences, USA) at 1:1 dilution were subcutaneously injected into the bilateral upper flank regions of the nude mice (3–4 weeks of age, female, BALB/c, six mice in each group). The tumor volumes were measured twice a week using Vernier Caliper. After 4 weeks, the mice were killed and the tumor volumes were measured by using the formula tumor volume (mm^3^)=(*L* × *W*^2^)/2, where L=long axis and W=short axis1. For metastatic model, Anti-miR-181d or vector overexpressed HCT116 (1 × 10^6^) cells were injected through lateral tail vein of the nude mice (six mice in each group), and after 4 weeks, the mice were killed and the number of nodules in the lung was counted and the lung tissue was stained with hematoxylin and eosin.

### Data analysis and statistics

All statistical analyses were resolved using the SPSS20.0 for Windows. All experiments were repeated at least three times, and results are presented either as one representative experiment or as an average±SD. Statistical analyses were performed using two-tailed Student’s *t* test or with two-way ANOVA. A *P*<0.05 was deemed to be statistically significant.

## Publisher’s Note

Springer Nature remains neutral with regard to jurisdictional claims in published maps and institutional affiliations.
