# Supplementary figures and images for: miR-181d and c-myc-mediated inhibition of CRY2 and FBXL3 reprograms metabolism in colorectal cancer
Source: Cell Death Dis. 2017 Jul 27;8(7):e2958–. doi: 10.1038/cddis.2017.300 (PMC5550850; doi:10.1038/cddis.2017.300)

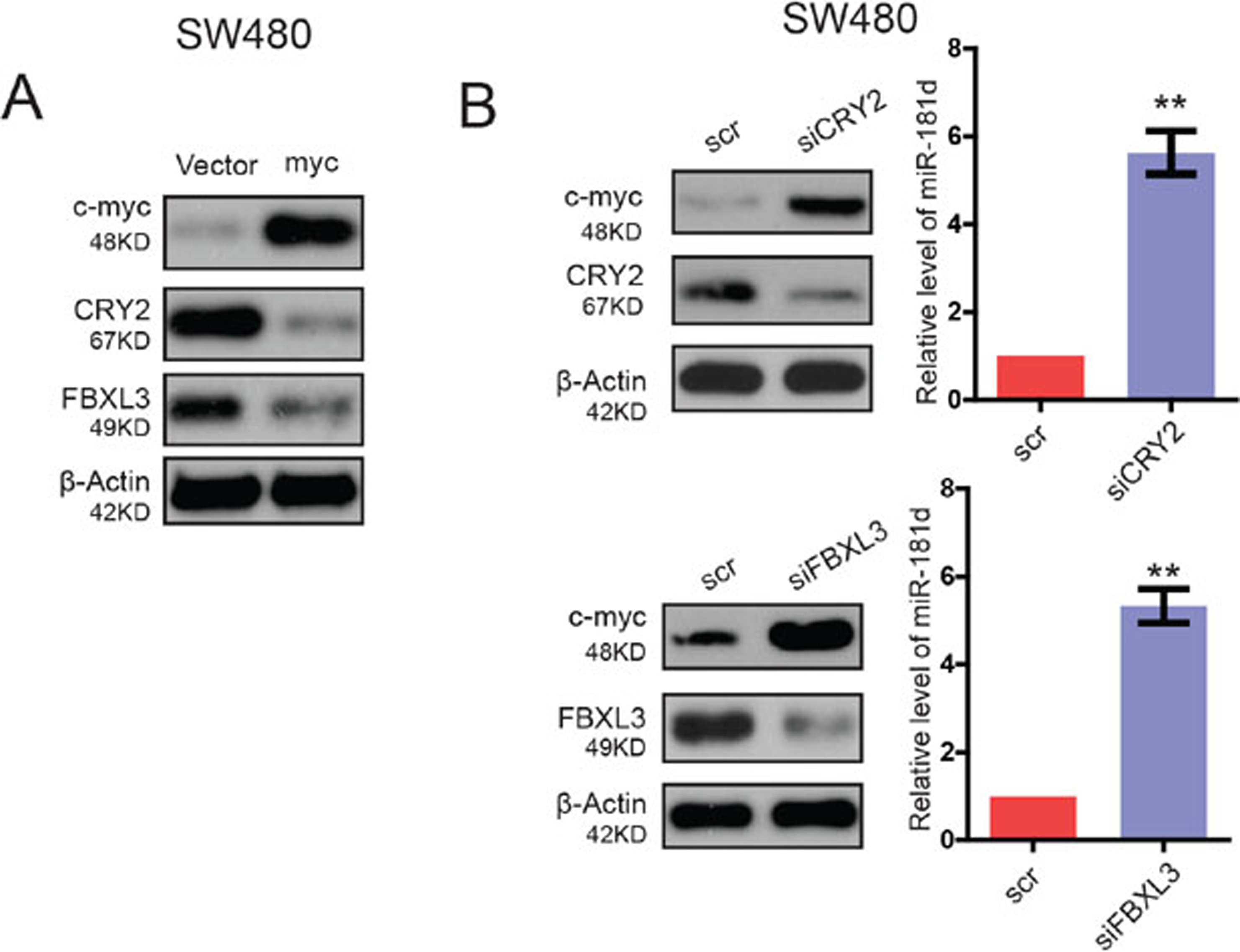

Supplement: Supplementary Figure S1 [file cddis2017300x1.tif]
