# Supplementary material for: miR-181d and c-myc-mediated inhibition of CRY2 and FBXL3 reprograms metabolism in colorectal cancer
Source: Cell Death Dis. 2017 Jul 27;8(7):e2958–. doi: 10.1038/cddis.2017.300 (PMC5550850; doi:10.1038/cddis.2017.300)
Supplement: Supplementary Figure Legend [file cddis2017300x2.docx]

**Supplementary Figure 1 The feedback regulation between c-myc,miR-181d, CRY2,and FBXL3 in CRC cells**.(**a**) The effect of overexpressing c-myc on the levels of CRY2 and FBXL3 in CRC cells. (**b**) The effect of knocking down the CRY2(upper panel) or FBXL3(lower panel). on the levels of c-myc and miR-181d in CRC cells. Protein expressions have been normalized using β-actin as internal control. qRT-PCR Data were normalized against U6.
